# Improving the Performance of Solution−Processed Quantum Dot Light−Emitting Diodes via a HfO_x_ Interfacial Layer

**DOI:** 10.3390/ma15248977

**Published:** 2022-12-15

**Authors:** Jun Hyung Jeong, Min Gye Kim, Jin Hyun Ma, Min Ho Park, Hyoun Ji Ha, Seong Jae Kang, Min-Jae Maeng, Young Duck Kim, Yongsup Park, Seong Jun Kang

**Affiliations:** 1 Department of Advanced Materials Engineering for Information and Electronics, Kyung Hee University, Yongin 17101, Republic of Korea; 2 Integrated Education Program for Frontier Materials (BK21 Four), Kyung Hee University, Yongin 17104, Republic of Korea; 3 Department of Physics and Research Institute of Basic Sciences, Kyung Hee University, Seoul 02447, Republic of Korea; 4 Department of Information Display, Kyung Hee University, Seoul 02447, Republic of Korea

**Keywords:** quantum dots, light−emitting diodes, solution process

## Abstract

One of the major obstacles in the way of high−performance quantum dot light−emitting diodes (QLEDs) is the charge imbalance arising from more efficient electron injection into the emission layer than the hole injection. In previous studies, a balanced charge injection was often achieved by lowering the electron injection efficiency; however, high performance next−generation QLEDs require the hole injection efficiency to be enhanced to the level of electron injection efficiency. Here, we introduce a solution−processed HfO_x_ layer for the enhanced hole injection efficiency. A large amount of oxygen vacancies in the HfO_x_ films creates gap states that lower the hole injection barrier between the anode and the emission layer, resulting in enhanced light−emitting characteristics. The insertion of the HfO_x_ layer increased the luminance of the device to 166,600 cd/m^2^, and the current efficiency and external quantum efficiency to 16.6 cd/A and 3.68%, respectively, compared with the values of 63,673 cd/m^2^, 7.37 cd/A, and 1.64% for the device without HfO_x_ layer. The enhanced light−emitting characteristics of the device were elucidated by X−ray photoelectron, ultra−violet photoelectron, and UV−visible spectroscopy. Our results suggest that the insertion of the HfO_x_ layer is a useful method for improving the light−emitting properties of QLEDs.

## 1. Introduction

Light−emitting diodes (LEDs) have been extensively researched in the past few decades as a component of electronic devices that have become indispensable for convenient human life. Various types of light−emitting materials, such as organics, perovskites, two−dimensional (2D) materials, and quantum dots (QDs) have been used to produce high brightness LEDs [1,2,3,4]. Among these light−emitting materials, QDs have been extensively emerging owing to their high color purity, solution processability, and facile tunable band gap [5,6].

Unfortunately, the brightness and efficiency of quantum dot light−emitting diodes (QLEDs) are not yet optimal and attempts were made to address these shortcomings by adjusting the charge imbalance between electron and hole injection [7,8,9]. The charge imbalance in QLEDs mainly originates from the presence of excess electrons and numerous studies have been conducted to resolve the problem. Recently, Jin et al. reported the inclusion of an organic electron−blocking layer (EBL) to rectify the charge balance by blocking the injection of electrons [10]. Zhang et al. reported the incorporation of a double−layered structure for electron transport to balance the charge injection [11].

Rather than restricting electron injection to balance the charge injection, another approach would be to improve the hole injection properties for high performance QLED devices. Accordingly, many efforts have been devoted to improve the hole injection characteristics of QLEDs, such as doping the hole injection layer (HIL) or hole transport layer (HTL). However, considering that the hole transporting characteristics are sensitively affected by the amount of dopant material, attaining efficient hole transporting characteristics with an undoped hole injection/transport layer continues to remain a challenge [12]. Attempts to subject the HIL or HTL to additional treatment have also been reported to improve the hole injection properties; however, this simultaneously complicated the device fabrication [13,14]. Therefore, introducing an additional anode buffer layer without doping or post−chemical treatment is necessary for obtaining solution−processed high performance QLEDs.

Here, we introduced a solution−processed HfO_x_ layer as an anode buffer layer to improve the performance of QLEDs. Although the HfO_x_ layer has been widely used as an insulating layer, modification thereof by introducing a large amount of defect states originating from the oxygen vacancies in the HfO_x_ film was expected to lower the hole injection barrier between the anode and emission layer. A large amount of oxygen vacancies was obtained via spin coating followed by thermally annealing the films at low temperature of 250 °C. The maximum luminance, external quantum efficiency, and current efficiency of the optimal device was 166,670 cd/m^2^, 3.68%, and 16.6 cd/A, respectively. These quantities, which are representative of the QLEDs performance, are more than two−fold higher than those of the device without the HfO_x_ layer, indicating that the HfO_x_ layer efficiently enhanced the performance of the QLEDs. 

## 2. Experimental Section

### 2.1. Solutions Preparation

Solutions of HfO_x_ with different concentrations were prepared by dissolving different amounts of HfCl_4_ (Sigma Aldrich, St. Louis, MO, USA) in 10 mL 2−methoxyethanol (Sigma Aldrich) to obtain 0.01, 0.03, and 0.05 M solution, after which the mixture was stirred for 30 min. A solution of vanadium oxide (V_2_O_5_) was prepared by dissolving 0.1 mL of vanadium triisopropoxide oxide (Alfa Aesar, Massachusetts, USA) in 13 mL of isopropanol (IPA) in a nitrogen−filled glove box. After vigorously stirring the solution for 30 min, 66.5 μL of deionized (DI) water was dropped into the solution to facilitate hydrolysis. A 1 wt% of Poly[(9,9−dioctylfluorenyl−2,7−diyl)−*co*−(4,4′−(4−*sec*−butylphenyl)diphenylamine)] (TFB) solution was prepared by dissolving TFB into *p*−xylene, followed by stirring at 80 °C for 30 min.

### 2.2. Device Fabrication

Patterned ITO glass was ultrasonicated with DI water, acetone, and IPA for 10 min. The substrates were dried in a stream of N_2_ gas, followed by exposure to UV−ozone for 15 min to render the surface hydrophilic and remove residual organics. Subsequently, the HfO_x_ solution was spin−coated onto the substrate at 3000 rpm for 30 s. The spin−coated HfO_x_ films were pre−annealed at 120 °C for 5 min to evaporate the solvents, and finally annealed at 250 °C for 45 min. After the coated substrate was allowed to cool, a V_2_O_5_ solution was spin−coated onto the HfO_x_ layer at 3000 rpm for 30 s, followed by annealing at 60 °C for 5 min. Next, a solution of TFB, the HTL material, was spin−coated onto the V_2_O_5_ layer at 3000 rpm for 30 s, followed by annealing at 180 °C for 30 min. Then, a solution of CdSe/ZnS QDs (Uniam, 20 mg/mL) was spin−coated onto the TFB layer at 2000 rpm for 30 s, followed by annealing at 100 °C for 10 min. The layer of QDs was covered with a layer of ZnO by spin coating a solution thereof (Avantama, N−10) onto the QDs at 2000 rpm for 30 s, followed by annealing at 100 °C for 10 min. Finally, an aluminum (Al) cathode (thickness: 130 nm) was thermally evaporated under vacuum (~8 × 10^−6^ Torr) at a rate of 3 A/s using a metal shadow mask with an illuminating area of 4 mm^2^.

### 2.3. Characterization

The electroluminescence characteristics of the QLEDs were measured using an OLED I−V−L test system (M6100, McScience Inc.,Suwon, Republic of Korea), which was equipped with a Keithley 2400 source meter and a Konica Minolta CS−2000 spectroradiometer. The ultra−violet photoelectron spectroscopy (UPS) measurements were performed at the Multidimensional Materials Research Center of Kyung Hee University using a helium discharge lamp (VG Scientific, photon energy 21.22 eV) in a custom−built ultra−high vacuum chamber (base pressure: 5 × 10^−10^ Torr). A sample bias of −10 V was applied to determine the secondary electron cut−off (SECO) positions when the work function (WF) was measured. All spectra were recorded at room temperature by using a hemispherical analyzer (Scienta SES100, Uppsala, Sweden). The overall energy resolution was approximately 0.1 eV. X−ray photoelectron spectroscopy (XPS) measurements were conducted in an ultrahigh vacuum chamber (~1 × 10^−9^ Torr), using a spectrometer (Nexsa, Thermo Fisher Scientific, Massachusetts, USA) with an Al−Kα (1486.6 eV) source. UV−visible measurements were conducted using a UV−vis spectrophotometer (Cary 100, Agilent, Santa Clara, USA) to acquire transmittance spectra of the layers. The surface morphology was investigated using AFM measurement (Dimension 3100 SPM, Bruker, Massachusetts, USA ).

## 3. Results and Discussion

**Figure 1:**
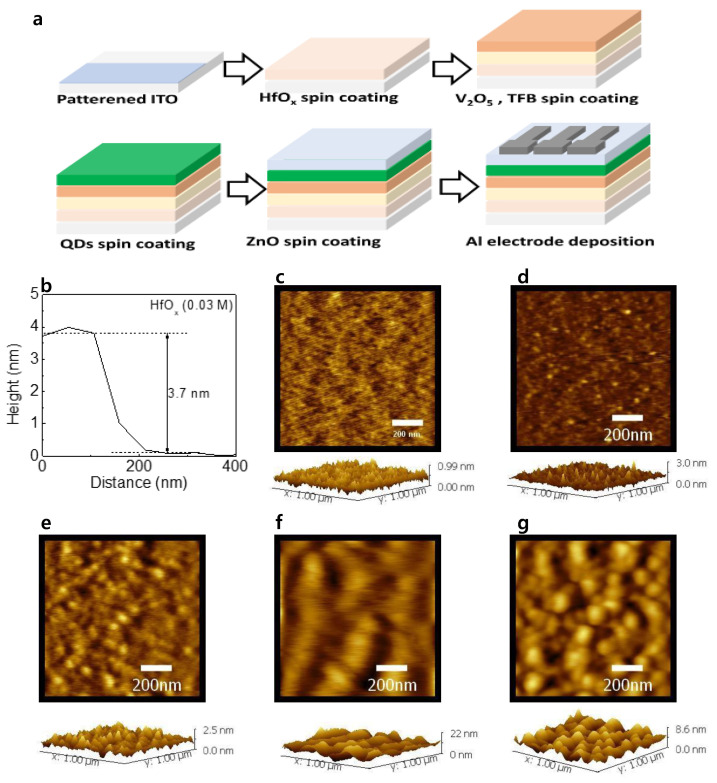
(**a**) Schematic illustration of the device fabrication. (**b**) Thickness of the HfO_x_ (0.03 M) films obtained from AFM measurement. Surface morphology with 3D topography of (**c**) HfO_x_ (0.03 M), (**d**) V_2_O_5_, (**e**)TFB, (**f**) QD, and (**g**) ZnO layers.

Figure 1a shows the schematic illustration of the device fabrication. The final structure of the device was ITO/(HfO_x_)/V_2_O_5_/TFB/QDs/ZnO/Al. Figure 1b shows the thickness of HfO_x_ (0.03 M) film obtained from AFM measurement. The thickness was obtained to be 3.7 nm. The thickness of V_2_O_5_, TFB, QDs, and ZnO can be estimated to be 4.5, 12, 131, and 38 nm from our previous report [15]. Figure 1c–g shows the surface morphology and 3D topography of HfO_x_ (0.03 M), V_2_O_5_, TFB, QD, and ZnO layers, respectively. The root mean square values of the layers were 0.1, 0.24, 0.31, 2.94, and 1.38 nm, respectively.

**Figure 2:**
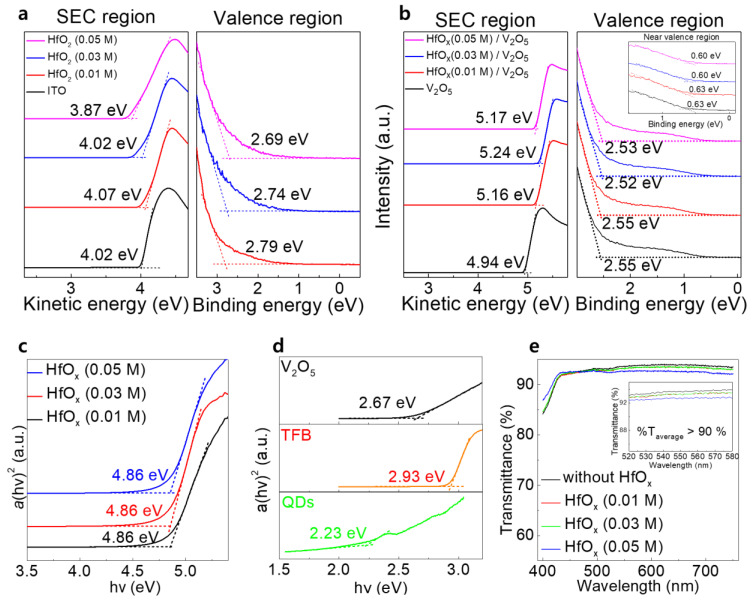
SEC and valence region spectra of (**a**) ITO, HfO_x_ (0.01 M), HfO_x_ (0.03 M), and HfO_x_ (0.05 M) films; and (**b**) V_2_O_5_, HfO_x_ (0.01 M)/V_2_O_5_, HfO_x_ (0.03 M)/V_2_O_5_, and HfO_x_ (0.05 M)/V_2_O_5_ films. The inset shows the near valence region spectra of each of the films. Tauc plots of the (**c**) HfO_x_ (0.01 M), HfO_x_ (0.03 M), and HfO_x_ (0.05 M) films; and (**d**) V_2_O_5_, TFB, and QDs. (**e**) Optical transmittance spectra of glass/V_2_O_5_/TFB/QDs/ZnO films with and without the HfO_x_ layer.

The UPS measurements were intended to determine the WF and valence band maximum (VBM) values of each of the layers from the SECO position and valence region. The SECO and valence region spectra of the ITO, ITO/HfO_x_ (0.01 M), ITO/HfO_x_ (0.03 M), and ITO/HfO_x_ (0.05 M) films are shown in Figure 2a. The obtained WF values of ITO, and ITO/HfO_x_ (0.01, 0.03, and 0.05 M) were 4.02, 4.07, 4.02, and 3.87 eV. The VBM values of HfO_x_ (0.01, 0.03, and 0.05 M) were found to be 3.40, 3.41, and 3.39 eV, respectively. The SECO and valence region spectra of V_2_O_5_, HfO_x_ (0.01 M)/V_2_O_5_, HfO_x_ (0.03 M)/V_2_O_5_, and HfO_x_ (0.05 M)/V_2_O_5_ are shown in Figure 2b. The inset shows the near valence region spectra of each of the V_2_O_5_ layers, indicating the gap states originating from the V^4+^ state of V_2_O_5_, as previously reported [16,17,18]. The WF values of V_2_O_5_ and those of the HfO_x_ (0.01, 0.03, and 0.05 M)/V_2_O_5_ layers were 4.94, 5.16, 5.24, and 5.17 eV, respectively. The VBM values of the V_2_O_5_ layers were 2.55, 2.55, 2.52, and 2.53 eV, respectively, and the differences between the Fermi level (E_F_) and gap state were 0.63, 0.61, 0.59, and 0.62 eV, respectively. Figure 2c shows the optical band gaps of the HfO_x_ films in the form of the Tauc plots derived from the UV−vis transmittance, using the following equation

(1) $$ahv=A{\left(hv-E_{g}\right)}^{n}$$

where α is the absorption coefficient, *hν* is the photon energy, A is a constant, *E_g_* is the optical band gap, and *n* = 1/2 for a direct band gap semiconductor [19]. All the HfO_x_ films had an optical band gap of 4.86 eV, regardless of the concentration of the HfO_x_ solution. Figure 2d shows the optical band gaps of the V_2_O_5_, TFB, and QD layers of 2.67, 2.93, and 2.23 eV, respectively, derived from the Tauc plot. The optical transmission spectra, recorded by conducting UV−vis measurements of the V_2_O_5_/TFB/QDs/ZnO films with and without the HfO_x_ layer, are shown in Figure 2e. All the films had high transmittance of more than 90% in the 520–580 nm region regardless of the presence of the HfO_x_ layer, indicating that the HfO_x_ films did not severely affect the transmittance of the device.

**Figure 3:**
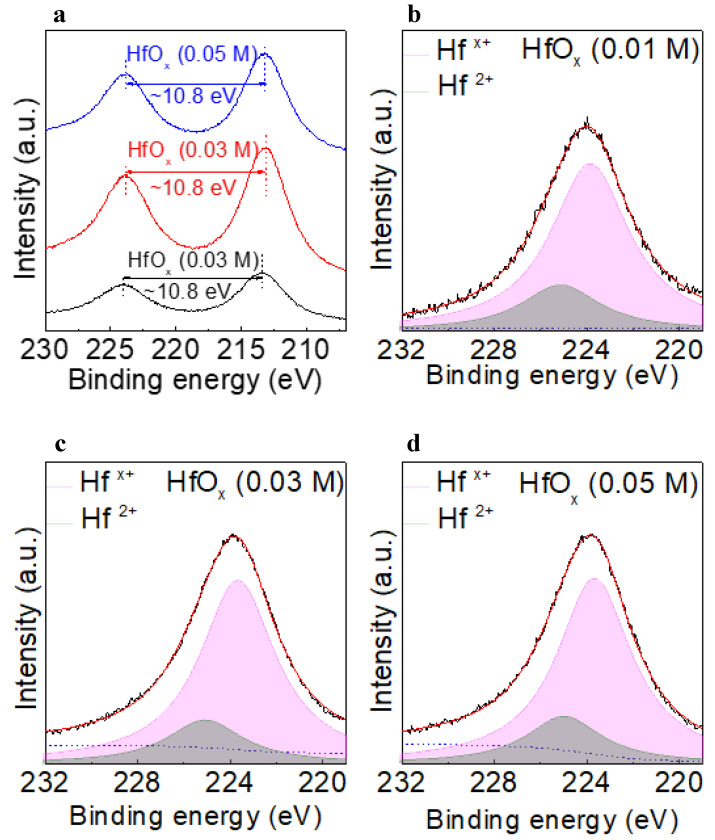
(**a**) Hf 4d spectra of HfO_x_ (0.01 M), HfO_x_ (0.03 M), and HfO_x_ (0.05 M) films. Deconvoluted Hf 4d_3/2_ XPS profiles of the (**b**) HfO_x_ (0.01 M), (**c**) HfO_x_ (0.03 M), and (**d**) HfO_x_ (0.05 M) films.

The chemical states of each of the layers in the device were examined in detail by using XPS to analyze for hafnium 4d (Hf 4d) and vanadium 2p (V 2p). Reliability was ensured by calibrating the binding energies by assigning the peak at 285 eV to C 1s. Figure 3a shows the Hf 4d spectra of the HfO_x_ (0.01 M), HfO_x_ (0.03 M), and HfO_x_ (0.05 M) films. The doublet at binding energies of ~224.2 eV and 213.4 eV arose as a result of spin orbit splitting of the Hf 4d orbitals into Hf 4d_3/2_ and Hf 4d_5/2_, respectively [20]. Figure 3b–d shows the Hf 4d_3/2_ XPS results for the HfO_x_ (0.01 M), HfO_x_ (0.03 M), and HfO_x_ (0.05 M) films, respectively. Because all the Hf 4d_3/2_ peaks were slightly asymmetric, as shown in Figure 3b−d, Hf ions of different valences were considered to exist in the HfO_x_ film. The XPS profile of Hf 4d_3/2_ was deconvoluted into two peaks using Gaussian–Lorentzian fitting. The asymmetric Hf 4d_3/2_ peak can be deconvoluted into two peaks centered at the binding energies of ~223.7 eV and ~225.1 eV. The Hf in HfO_x_ is widely known to exist in the Hf^4+^ and Hf^x+^ (x < 4) states. The lower electronegativity of Hf (1.3) than oxygen (3.44) suggests that the Hf with lower valency (Hf^x+^), which would be less affected by the surrounding oxygen atoms, and would have lower binding energy than stoichiometric Hf in the form of HfO_2_ [21]. This suggests that the amount of Hf^x+^ is related to the amount of oxygen vacancies in the film, which is consistent with our previous report [22]. The concentration of Hf^x+^ in HfO_x_ (0.01 M), HfO_x_ (0.03 M), and HfO_x_ (0.05 M) was 78.4, 80.7, and 79.5%, respectively, indicating that the HfO_x_ (0.03 M) film possesses the highest amount of oxygen vacancies. 

**Figure 4:**
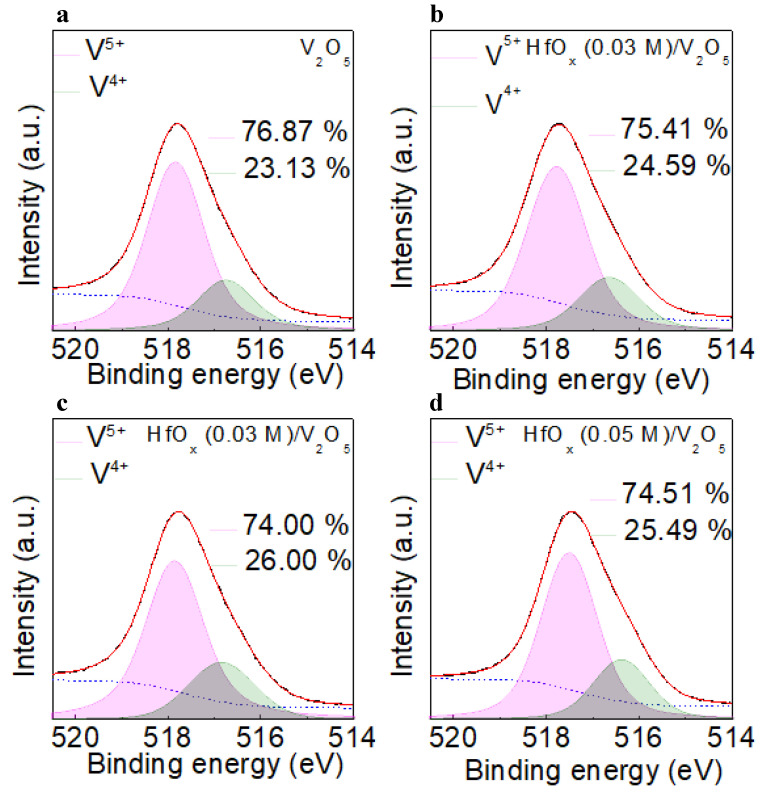
Deconvoluted V 2p_3/2_ XPS results of the (**a**)V_2_O_5_, (**b**) HfO_x_ (0.01 M)/V_2_O_5_, (**c**) HfO_x_ (0.03 M)/V_2_O_5_, and (**d**) HfO_x_ (0.05 M)/V_2_O_5_ films.

Figure 4a−d shows the deconvoluted V 2p_3/2_ XPS profiles for the V_2_O_5_, and HfO_x_ (0.01, 0.03, and 0.05 M)/V_2_O_5_ films, respectively. The V 2p_3/2_ spectrum was deconvoluted into two peaks centered at the higher and lower binding energies of ~517.7 eV and ~516.6 eV, respectively. The peak centered at the higher of these two values indicates the V in V^5+^, whereas the peak centered at the lower value indicates the V in V^4+^ [23]. Because the V^4+^ state forms a hole transport channel near the Fermi level, it is necessary to clarify the concentration of V^4+^ in the V_2_O_5_ film [16]. The concentration of V^4+^ in V_2_O_5_, and in the HfO_x_ (0.01, 0.03, and 0.05 M)/V_2_O_5_ films was 23.13, 24.59, 26.00, and 25,49%; thus, the HfO_x_ (0.03 M)/V_2_O_5_ film had the highest concentration of V^4+^. During the formation of the V_2_O_5_ layer above the oxygen−deficient HfO_x_ layer, the oxygen can diffuse from V_2_O_5_ to HfO_x_ because of the difference in the oxygen density between V_2_O_5_ and HfO_x_. This indicates that the highest V^4+^ concentration of HfO_x_ (0.03 M)/V_2_O_5_ could be the consequence of the existence of the highest concentration of oxygen vacancies in HfO_x_ (0.03 M) film [24]. 

**Figure 5:**
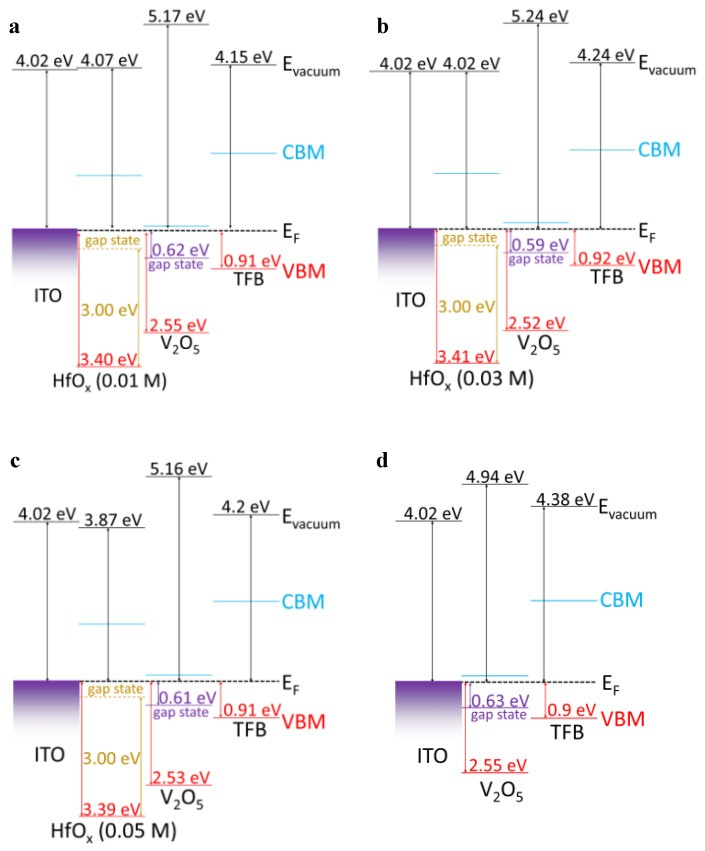
Schematic energy level diagram of QLEDs (**a**) without the HfO_x_ layer, and with the (**b**) HfO_x_ (0.01 M), (**c**) HfO_x_ (0.03 M), and (**d**) HfO_x_ (0.05 M) layer.

Figure 5 shows the schematic interfacial energy band diagram of the QLEDs with the aligned Fermi level corresponding to the UPS results and the Tauc plot. The V^4+^−related gap states of V_2_O_5_ near the Fermi level are shown in Figure 5a–d. In the case of HfO_x_, Hadacek et al. and Hildebrandt et al. reported that the oxygen−deficient HfO_x_ films exhibit *p*−type behavior. In addition, Kaisar et al. revealed that the origin of the *p*−type conductivity of oxygen−deficient HfO_x_ is due to the oxygen vacancy−related defect states, which are located ~3 eV above the VBM [25,26,27]. Because of the high concentration of Hf^x+^ in our HfO_x_ films representative of the large amount of oxygen vacancies in the HfO_x_ films, we indicated that the oxygen−related defect states of HfO_x_ are located 3 eV above the VBM. Therefore, as shown in Figure 5, all the devices containing the HfO_x_ layer would be expected to exhibit enhanced hole injection characteristics owing to the lowered hole injection barrier. In addition, because the device with the HfO_x_ (0.03 M) layer has the lowest hole injection barrier of 0.18 eV at the HfO_x_/V_2_O_5_ interface, the device with the HfO_x_ (0.03 M) layer would be predicted to exhibit the optimal hole injection characteristics.

**Figure 6:**
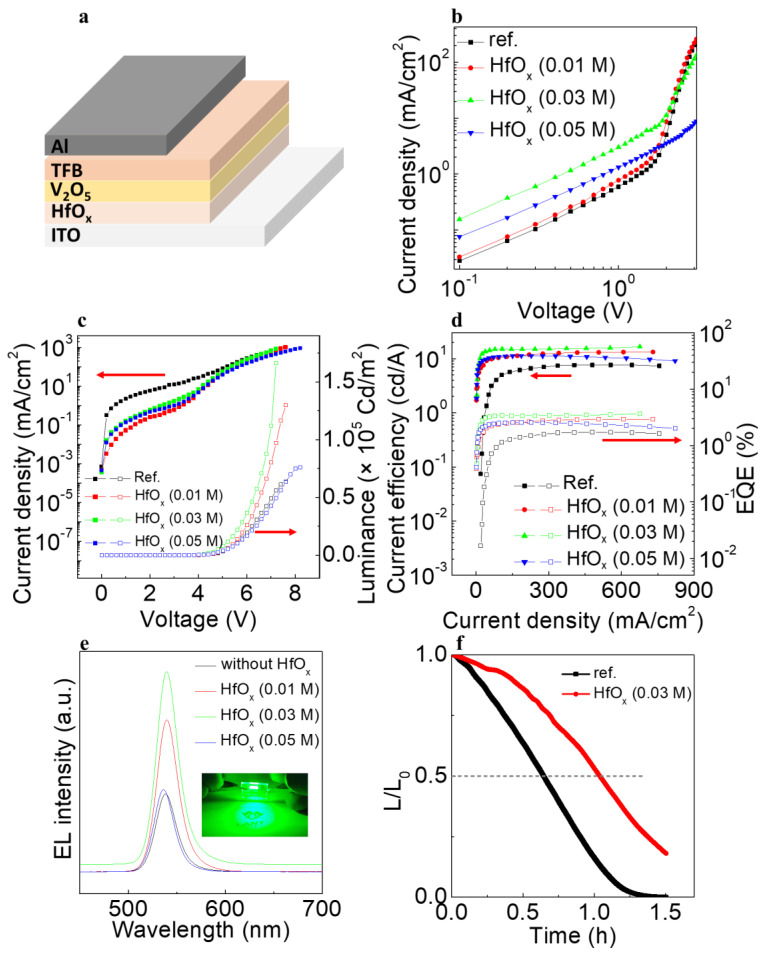
(**a**) Schematic illustration of the device fabrication process (**b**) J−V characteristics of HOD devices. (**c**) J−V−L characteristics and (**d**) current efficiency−current density plot of the QLED devices. (**e**) EL spectra of the QLED devices at each maximum luminance. (**f**) Lifetime data of the QLED devices without and with HfO_x_ (0.03 M) layer.

To corroborate the abovementioned results, we first recorded the current density−voltage (J−V) curves of the hole only devices (HODs) with the structure ITO/(HfO_x_)/V_2_O_5_/TFB/Al as shown in Figure 6a−b. The HOD without the HfO_x_ film had the lowest current density, with that of the HfO_x_ (0.01 M) film being slightly higher. The HOD with the HfO_x_ (0.03 M) film had the highest current density. Considering the alignment of the interfacial energy bands, as shown in Figure 5, the enhanced current density of the HOD can be attributed to the well−aligned gap states between the HfO_x_ and V_2_O_5_ layers. Moreover, taking into account that the HfO_x_ (0.03 M)/V_2_O_5_ film contains the highest concentration of gap states, originating from the V^4+^ state, the well−aligned energy levels and the concentration of gap states can both contribute to efficient hole injection. Figure 6c shows the current density−voltage−luminance (J−V−L) characteristics of the QLEDs with and without the HfO_x_ (0.01, 0.03, and 0.05 M) layer. Consistent with the aforementioned improved hole injection characteristics, the QLED with the HfO_x_ (0.03 M) layer had the highest luminance of 166,670 cd/m^2^, more than twice as high as the QLED without the HfO_x_ layer. In terms of the current density, the device without the HfO_x_ layer had higher values compared with the devices with the HfO_x_ layer. This can be attributed to the high conduction band offset between HfO_x_ and V_2_O_5_, which would have efficiently blocked the leakage current toward the ITO. Owing to the diminished leakage current and enhanced hole injection characteristics, the current efficiency (CE) and external quantum efficiency (EQE) of QLEDs with the HfO_x_ (0.03 M) layer increased considerably, as shown in Figure 6c,d. The device with the HfO_x_ (0.03 M) layer exhibited CE and EQE values of 16.6 cd/A and 3.68%, which is more than two−fold higher than the 7.37 cd/A and 1.64% of the device without the HfO_x_ layer. Figure 6e shows the electroluminescence (EL) spectra of the QLED devices with the HfO_x_ (0.01 M), HfO_x_ (0.03 M), and HfO_x_ (0.05 M) layers, and without the HfO_x_ layer at the maximum luminance of each of these devices. The operating image of the QLED is shown in the inset of Figure 6e. All the QLEDs had an emission peak with full width at half maximum (FWHM) of ~25 nm without significant peak shift, indicating that the devices exhibited high color purity. To identify the stability of the device, the lifetime was confirmed at which the luminance reached half of the initial value with the initial luminance discussed, as shown in Figure 6f. The initial luminance values of the devices without HfO_x_ and with HfO_x_ (0.03 M) were 2320.2 and 2535.9 cd/m^2^, respectively. The lifetimes of the devices at an *L*_0_ = 100 cd/m^2^ was obtained using the following equation

(2) $${\left(L_{0}\right)}^{n}\times T_{50}=constant$$

where *n* is the acceleration factor (1.514), L_0_ is the initial luminance, L is the luminance, and T_50_ is the time which the luminance reached the half of the initial luminance value [22]. The longer lifetime of 139 h was obtained by adding HfO_x_ (0.03 M) layer, compared with the 75.9 h of the device without HfO_x_ layer.

## 4. Conclusions

We fabricated QLEDs with highly enhanced light−emitting characteristics by adding a solution−processed HfO_x_ interfacial layer. The defect states of the HfO_x_ layer, originating from the large amount of oxygen vacancies, could be well−aligned with the gap state of V_2_O_5_, resulting in lowering the hole injection barrier from ITO to V_2_O_5_. Furthermore, the oxygen−deficient HfO_x_ film also contributed to the increase in the formation of the V^4+^ state in the V_2_O_5_ layer, resulting in enhanced hole injection characteristics. Owing to the improved hole injection characteristics, the device with the optimal HfO_x_ concentration had the highest luminance of 166,670 cd/m^2^, current efficiency of 16.6 cd/m^2^, and EQE of 3.68% compared with the values of 63,673 cd/m^2^, 7.37 cd/A, and 1.64%, respectively, for the device without HfO_x_ layer. UPS and XPS measurements enabled us to identify the origin of the enhanced light−emitting characteristics of the devices. Our results indicate that we developed a useful and facile method for improving the hole injection characteristics of QLEDs by incorporating a solution−processable HfO_x_ interfacial layer.

## Data Availability

Data will be made available from the corresponding authors on reasonable request.
